# Inhibition of Astrocyte Reactivity by Mdivi-1 After Status Epilepticus in Rats Exacerbates Microglia-Mediated Neuroinflammation and Impairs Limbic–Cortical Glucose Metabolism

**DOI:** 10.3390/biom15091242

**Published:** 2025-08-27

**Authors:** Francisca Gómez-Oliver, Rubén Fernández de la Rosa, Mirjam Brackhan, Pablo Bascuñana, Miguel Ángel Pozo, Luis García-García

**Affiliations:** 1 Unidad de Cartografía Cerebral, Instituto Pluridisciplinar, Universidad Complutense de Madrid, 28040 Madrid, Spain; gomezof@ucm.es (F.G.-O.); rufernan@ucm.es (R.F.d.l.R.); brackhanm@gmail.com (M.B.); pablo.bascunana@salud.madrid.org (P.B.); pozo@ucm.es (M.Á.P.); 2 Departamento de Farmacología, Farmacognosia y Botánica, Facultad de Farmacia, Universidad Complutense de Madrid, 28040 Madrid, Spain; 3 Instituto de Investigación Sanitaria San Carlos (IdISSC), Hospital Clínico San Carlos, 28040 Madrid, Spain; 4 ICTS Bioimagen Complutense (BIOIMAC), Universidad Complutense de Madrid, 28040 Madrid, Spain; 5 Departamento de Fisiología, Facultad de Medicina, Universidad Complutense de Madrid, 28040 Madrid, Spain

**Keywords:** lithium–pilocarpine model, status epilepticus, Mdivi-1, 2-deoxy-2-[^18^F]fluoro-D-glucose ([^18^F]FDG), positron emission tomography (PET), hypometabolism, astrocyte, microglia, neuroinflammation

## Abstract

The lithium–pilocarpine rat model of status epilepticus (SE) is a well-established paradigm for studying epileptogenesis. Astrocyte reactivity has been implicated in modulating seizure susceptibility and neuroinflammation, yet its functional role in early epileptogenesis remains unclear. Herein, we evaluated the effects of Mdivi-1, a pharmacological inhibitor of mitochondrial fission protein Drp1, for its ability to modulate astrocytic mitochondrial dynamics and for its reported preventive neuroprotective properties. Mdivi-1 was administered shortly after SE onset, and we assessed brain glucose metabolism using [^18^F]FDG PET, alongside histological markers of neurodegeneration, astrocyte reactivity, and microglial activation, at 3 days post-SE. As expected, SE induced widespread brain hypometabolism measured by a VOI analysis, hippocampal neurodegeneration, and glial activation. Post-SE Mdivi-1 administration reduced hippocampal astrogliosis but neither conferred neuroprotection nor rescued glucose metabolism. On the contrary, Mdivi-1 exacerbated limbic–cortical hypometabolism when evaluated by SPM and normalized to whole brain tracer uptake and microglia-mediated neuroinflammation. These findings challenge the assumption that early astrocyte inhibition confers neuroprotection. Furthermore, early suppression of astrocyte reactivity after the damage has occurred may shift the neuroinflammatory response toward maladaptive microglial activation. Thus, while Mdivi-1 holds promise as a preventive neuroprotective agent, its use post-SE may have unintended adverse effects on the brain’s response to SE.

## 1. Introduction

Epilepsy is one of the most prevalent chronic neurological disorders, defined by a predisposition to generate spontaneous recurrent seizures (SRSs) and often associated with disabling consequences [1]. Among its various forms, temporal lobe epilepsy (TLE) is the most common focal epilepsy in adults and frequently exhibits resistance to conventional antiepileptic pharmacotherapy [2]. TLE is commonly linked to hippocampal sclerosis [3] and interictal glucose hypometabolism localized to the epileptogenic zone [4,5], which can be reliably visualized using 2-deoxy-2-[^18^F]fluoro-D-glucose positron emission tomography ([^18^F]FDG-PET). Notably, interictal focal hypometabolism identified via PET imaging is regarded as one of the most robust early biomarkers of TLE [6,7].

The lithium–pilocarpine model of status epilepticus (SE) in rodents is extensively employed to study epileptogenesis, as it replicates many neuropathological and behavioral features of human TLE [8,9,10]. Following pilocarpine-induced SE, animals enter a latent phase characterized by widespread cerebral hypometabolism [11,12,13,14,15,16,17]. This hypometabolic state is a recognized early biomarker of epileptogenesis [15,18] and coincides with key pathological events such as neurodegeneration, neuronal loss, neuroinflammation, and reactive gliosis [13,14,16,17,19]. The metabolic dysfunction underlying this phase is thought to result from a combination of neuronal death, a reduced synaptic density [4,5], and impaired cerebral perfusion [20].

Mdivi-1, a selective inhibitor of the mitochondrial fission protein dynamin-related protein 1 (Drp1), has emerged as a promising neuroprotective compound. While originally explored for its anticancer potential, including demonstrated efficacy in various cancer models [21], its application in the context of neurological disorders has garnered growing interest.

Although Mdivi-1 has not yet been approved for clinical use, preclinical studies have consistently shown its neuroprotective efficacy across multiple models of neurological disorders. Thus, Mdivi-1 has been shown to improve neurological outcomes in experimental models of ischemic stroke, traumatic brain injury, and neurodegenerative diseases such as Alzheimer’s and Parkinson’s disease [22], as well as in different animal models of epileptogenesis [23,24,25,26,27,28]. These protective effects are primarily attributed to its modulation of mitochondrial dynamics, particularly through the inhibition of excessive mitochondrial fission, a process often linked to neuronal injury and energy dysfunction.

Beyond fission inhibition, Mdivi-1 also enhances mitochondrial biogenesis and supports energy metabolism, thereby preserving neuronal function in various disease models [29,30,31]. Through these mechanisms, Mdivi-1 helps to maintain mitochondrial integrity, suppress apoptosis-related signaling, and attenuate oxidative stress, which are factors critically involved in both acute and chronic neurological damage.

Mdivi-1 is widely employed as a pharmacological tool to investigate mitochondria-dependent stress responses, particularly in astrocytes, through the inhibition of Drp1 [32,33]. However, growing evidence highlights important limitations that warrant caution in interpreting its effects, particularly due to its limited cellular and molecular specificity [34,35,36]. Thus, Mdivi-1’s primary mechanism, selective inhibition of Drp1, affects multiple cell types, and astrocytes appear particularly sensitive due to their dynamic mitochondrial behavior and central roles in neuroinflammation and scar formation. This context-dependent responsiveness positions Mdivi-1 as a promising modulator of astrocyte-driven pathology in central nervous system injuries.

Despite its promising profile, the neuroprotective potential of Mdivi-1 remains underexplored in the context of acquired epilepsy. In particular, its efficacy in the lithium–pilocarpine model of SE has yet to be systematically investigated. Moreover, to our knowledge, no studies have examined the effects of Mdivi-1 on cerebral glucose metabolism, a critical marker of neuronal activity and viability.

However, it is noteworthy that the previously mentioned studies reporting neuroprotective effects of Mdivi-1 have typically involved preventive administration prior to injury onset [23,24,25,26,27,28]. Considering that (i) this does not reflect the clinical reality where treatment is often initiated after a damaging event has occurred and (ii) in the context of SE, the hours and days following the insult are marked by profound glial activation, oxidative stress, mitochondrial dysfunction, and widespread metabolic disruption, the present study aimed to evaluate the short-term effects of Mdivi-1 when administered immediately following SE. Thus, we studied the effects on brain glucose metabolism, using [^18^F]FDG-PET imaging. Additionally, we assessed the effects of Midivi-1 on histopathological markers of neuroprotection and neuroinflammation in order to better understand its therapeutic potential in the early stages of epileptogenesis.

## 2. Materials and Methods

### 2.1. Animals

Forty adult male Sprague–Dawley rats (Charles River, Sant Cugat del Vallès, Barcelona, Spain), weighing 378.9 ± 10.5 g at the start of the experiment, were used. Animals were housed in pairs in standard ventilated cages (Tecniplast, Buguggiate, Italy) at controlled temperature (22 ± 2 °C) on a 12 h light/dark cycle (8:00 a.m. to 8:00 p.m.). Rats were allowed at least 5 days to acclimate to the new environment prior to any procedures. Environmental enrichment included chew sticks and plastic tunnels. Food and water were available ad libitum, except for a 12 h fasting period prior to the PET imaging acquisitions.

All procedures complied with European (2010/63/EU; http://data.europa.eu/eli/dir/2010/63/oj, accessed on 15 September 2024) and Spanish (RD53/2013; https://www.boe.es/eli/es/rd/2013/02/01/53, accessed on 15 September 2024) regulations for animal welfare. This study was approved by the Animal Research Ethics Committee of the Complutense University of Madrid and the Autonomous Community of Madrid (PROEX 222.1/20, 16 July 2020). All efforts were made to minimize animal suffering.

### 2.2. Lithium–Pilocarpine SE Model and Mdivi-1 Administration

**Figure 1:**
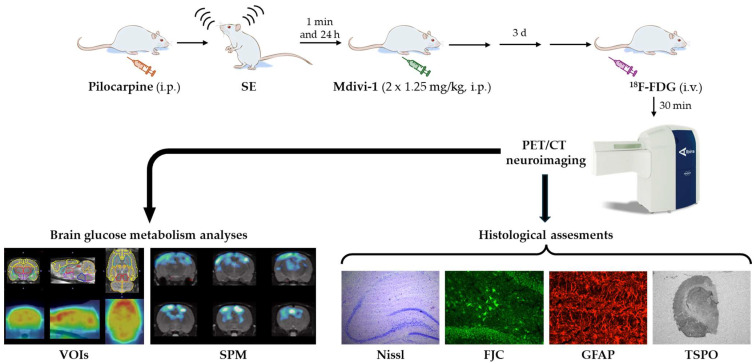
Schematic representation depicting the experimental design and procedures.

The experimental design is summarized in Figure 1. The lithium–pilocarpine model of SE was induced as previously described [13,14,16,17]. Briefly, rats received lithium chloride (127 mg/kg, i.p.; Merck-Sigma–Aldrich, Darmstadt, Germany) 18–20 h prior to pilocarpine administration (25 mg/kg, i.p.; Sigma–Aldrich). Thirty minutes before the pilocarpine injection, the animals were pretreated with methyl-scopolamine (2 mg/kg, i.p.) to reduce peripheral cholinergic effects.

SE onset was defined as Racine stage 4 (rearing with forelimb clonus) with continuous seizure activity [37]. After 45 min, pentobarbital (25 mg/kg, i.p.) was administered to terminate seizures. Rats that failed to reach stage 4 were excluded from this study. In case seizure behavior persists for 60 min after the pentobarbital injection, a half-dose of pentobarbital was administered. Control rats received the same treatment schedule but with saline (1 mL/kg, i.p.).

Mdivi-1 (1.25 mg/kg, i.p.; Merck-Sigma–Aldrich, Darmstadt, Germany) dissolved in 25% dimethyl sulfoxide (DMSO) in sterile 0.9% NaCl was administered at two time points: 1 min and 24 h after SE induction. The selected dose, vehicle, and route of administration were based on previous reports demonstrating both Dpr1-related modulation and neuroprotective effects on the pilocarpine-induced seizures in rats [24,25,26].

Thus, the experimental groups were as follows: (i) SAL + VEH (n = 9); (ii) PILO + VEH (n = 11); (iii) SAL + MDIVI (n = 9); and (iv) PILO + MDIVI (n = 11).

### 2.3. Body Weight (BW) Measurements

Rats were weighed at five time points throughout the study: 24 h before the pilocarpine injection (before the lithium chloride injection), at the pilocarpine injection, and 24 h, 48 h, and 72 h after the pilocarpine injection. Following PET scanning, animals were sacrificed by decapitation and brains were collected for histological analysis.

### 2.4. [^18^F]FDG PET/CT Imaging

[^18^F]FDG PET scans were acquired at 3 days post-SE, during the silent period, following the protocol established in our lab [13,14,16,17,38]. Briefly, the radiotracer ([^18^F]FDG; ~13 MBq in 0.2 mL of saline; Curium Pharma, Madrid, Spain) was injected via tail vein. Immediately after the injection, the rats were returned to their cages for a 30 min period to ensure the brain uptake of the radiotracer. After this awake uptake period, animals were anesthetized with isoflurane/oxygen and scanned using a dual PET/CT scanner (Albira PET/CT, Bruker NMI, Karlsruhe, Germany). The images acquisitions consisted of a 20 min static PET scan followed by a high-resolution computed tomography (CT) scan. Once acquired, the PET images were reconstructed using a Maximum Likelihood Expectation Maximization (MLEM) algorithm, applying decay, random, and scatter corrections. The CT images were reconstructed by the Filtered Back Projection (FBP) algorithm. For both reconstruction procedures, the own Albira embedded software was used (Albira Suite 5.8, Bruker NMI, Karlsruhe, Germany).

#### 2.4.1. PET Image Analysis by Volume-of-Interest (VOI) Analysis

Each individual CT skull image was manually co-registered to a magnetic resonance imaging (MRI) rat brain template [39] to obtain a fitted spatial mathematical transformation. Later, such a mathematical transformation was applied to the corresponding PET image, thus correcting and matching the PET image and the MRI template (as previously described) [40]. This co-registered PET image was overlayed onto the MRI template (containing the pre-defined brain areas) to quantify the tracer’s regional uptake values (kBq/mL). Furthermore, normalization to standardized uptake value (SUV; g/mL) was performed correcting by the animal BW, the injected dose, and decay of the radiotracer using the following formula: [Tracer uptake (kBq/mL) × BW (g))/(Tracer dose administered (kBq))]. These procedures were performed using PMOD 4.1 (PMOD Technologies Ltd., Zurich, Switzerland).

#### 2.4.2. PET Evaluation by Statistical Parametric Mapping (SPM)

Very often in experimental PET studies, the brain regions affected by the condition under investigation are not known beforehand and may not align with predefined anatomical VOIs. Unlike conventional VOI-based quantification (see Section 2.5), SPM performs a whole-brain analysis without prior assumptions about which areas are involved in the experimental condition [41]. This method can reveal changes in specific subregions that might be overlooked by the VOI analysis. Additionally, normalizing regional uptake to the entire brain enables the assessment of relative changes across regions, minimizing the influence of variations in cerebral blood flow or the peripheral tracer distribution.

SPM analyses were conducted using MATLAB v9.7 (The MathWorks, Natick, MA, USA) and the SPM12 toolbox (Wellcome Trust Center for Neuroimaging, UCL, London, UK), following previously described procedures [38]. To assess the impact of Mdivi-1 treatment on SE-induced hypometabolism, statistical comparisons between the PILO + VEH and PILO + MDIVI-1 groups were performed using *t*-tests. A *t*-value threshold corresponding to *p* < 0.05 was applied to identify significant voxels. The resulting parametric t-maps were imported into PMOD and co-registered with a rat brain T2-weighted MRI template.

### 2.5. Brain Collection and Processing for Neurohistochemical Studies

After PET scans, the rats were sacrificed, and their brains were dissected and divided into two hemispheres. The resulting hemibrains were immediately frozen on dry ice and later stored at −80 °C until the day of slicing. Coronal sections (25 μm thick) containing the dorsal hippocampus were obtained using a cryostat (Leica CM1850, Leica Biosystems, Nubloch, Germany) and thaw-mounted onto Superfrost Plus slides (Thermo Scientific, Dreieich, Germany). Slices were dried on a hot plate at 37 °C for approximately 5 min and then stored at −80 °C until further analysis. In order to prevent any potential bias or alteration, all samples were processed concurrently under identical conditions during all assessments.

### 2.6. Nissl Staining

For the qualitative assessment of neuronal viability and hippocampal integrity, Nissl staining with cresyl violet was performed as previously described [13,16,42]. Briefly, the slices were fixed for 10 min with 4% formaldehyde dissolved in phosphate buffer, pH 7.4, washed, and stained for 30 min with a solution containing 0.5% cresyl violet acetate. Afterwards, the sections were washed and dehydrated in a series of increasing ethanol concentrations (70%, 95%, and 100%). Finally, the slices were cleared with a non-toxic xylene substitute (Merck-Sigma–Aldrich, Darmstadt, Germany) and mounted with DPX (Herter, Barcelona, Spain). Images were obtained using a color digital camera Leica DFC425 (Leica Biosystems, Nubloch, Germany) coupled to a DM 2000 LED microscope with Leica Application Suite 4.7 software.

### 2.7. Fluoro-Jade C (FJC) Labeling

Hippocampal neurodegeneration was assessed following the protocol previously described [38,43] using FJC (0.0001% in PBS, 2 min; Millipore, Darmstadt, Germany), which requires unfixed tissue. After incubation, the sections were dried and mounted with DPX mounting media (Herter, Barcelona, Spain). Images were collected with a Leica DFC3000G camera using a fluorescein isothiocyanate (FITC) filter coupled to a microscope (Leica DM 2000 LED, Leica Biosystems, Nubloch, Germany). Neurodegeneration was evaluated by counting the number of FJC positive-neurons in the hippocampal subregions CA1, CA3, and hilus using the semi-automatic “Analyze Particles Function” available in the ImageJ 1.54g software (developed at the National Institutes of Health (NIH) and freely available on https://imagej.net/ij/download.html, accessed on 11 June 2025).

### 2.8. Reactive Astrogliosis Analysis by Glial Fibrillary Acidic Protein (GFAP) Immunofluorescence Staining

Astrogliosis was assessed by one-step GFAP immunofluorescence staining using a previously reported protocol [13,14,16,17,38]. Briefly, sections were fixed with 4% formaldehyde, blocked, permeabilized with a solution containing 3% BSA and 0.1% Triton X-100 in TBS (60 min), and incubated overnight with anti-GFAP-Cy3 (1:500, Sigma–Aldrich) in 1% BSA in TBS at 4 °C. The next day, the samples were washed with 0.1% Tween 20 diluted in TBS (3 × 5 min) and then mounted with Mowiol. Micrograph images were captured and evaluated using the same optical systems used for FJC staining but using a tetramethylrhodamine (TRITC) filter. GFAP fluorescence intensity in the dorsal hippocampus was quantified with ImageJ 1.54g software and expressed as a percentage relative to the SAL + VEH control group.

### 2.9. Analysis of Microglia-Mediated Neuroinflammation by [^3^H]PK11195 Autoradiography

To evaluate microglial activation, translocator protein (TSPO; 18 kDa) expression was assessed using [^3^H]PK11195 (Perkin Elmer, Rodgau, Germany) autoradiography following a modified protocol, as previously reported [14,16,17,38]. sections were dried (37 °C, 10 min), preincubated with 50 mM Tris-HCl (pH 7.4, 15 min), and then incubated with 1 nM [^3^H]PK11195 for 60 min. After ice-cold distilled water washes (2 × 5 min), sections were dried and exposed to autoradiographic film (Kodak BioMax MR, Carestream, Rochester, NY, USA) for approximately 2 months. Developed films were placed on a light box (Kaiser Prolite 5000, Kaiser Fototechnik, Buchen, Germany) and the autoradiographic images were captured with a Leica DFC425 camera coupled to a Leica MZ6 stereomicroscope. Both the delineation and quantification steps were run using ImageJ software version 1.54g. Optical density (O.D.) values were used to estimate neuroinflammation levels.

### 2.10. Statistical Analyses

The data are shown as means ± standard errors of the means (SEMs). Analyses were performed with SigmaPlot 11.0 software (Systat Software Inc., San Jose, CA, USA). Prior to conducting the parametric tests, the data were tested for normality and equal variance to ensure that the assumptions of the analysis were met. The mortality rate was only analyzed by comparing the PILO + VEH and PILO + MDIVI groups with a z-test for rates and proportions. BW, PET, and neurohistochemical data were analyzed by two-way analysis of variance (ANOVA), with pilocarpine and Mdivi-1 treatments as the two main factors. The effects of Mdivi-1 on the number of FJC+ neurons in pilocarpine-treated rats were compared in each hippocampal subregion and were assessed by Student’s *t*-test. When the interaction between factors was significant, further post hoc Tukey tests were performed. Statistical significance was considered when *p* < 0.05.

## 3. Results

### 3.1. Mortality Rate

**Figure 2:**
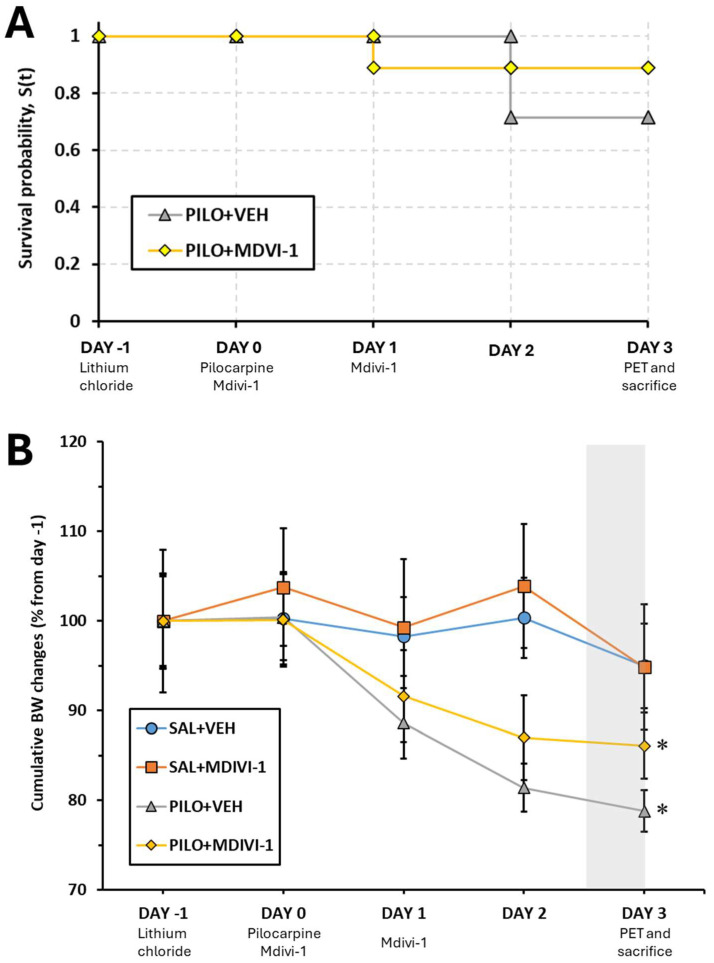
The administration of Mdivi-1 (1 min and 24 h after SE) had no significant effect on SE-induced mortality or BW loss in the rat lithium–pilocarpine model. (**A**) Kaplan–Meier curve depicting the survival probability, S(t), of the two experimental groups subjected to lithium–pilocarpine SE modeling. (**B**) Cumulative BW changes throughout the experimental period ranging from the day of LiCl administration (day −1) to the day of PET/CT scanning and sacrifice (day 3) are shown. BW changes were calculated as a percentage of the initial BW (day −1). The gray background area reflects the 12 h fasting period prior to the [^18^F]FDG PET/CT procedures. The data are shown as means ± SEMs (n = 7–10 rats/group, rats that survived the experimental procedure). Significant differences are depicted as * *p* < 0.05 compared with the PILO + VEH group, two-way ANOVA followed by post hoc Tukey tests.

The mortality rate following SE was 33.4% (four out of eleven rats) in the PILO + VEH group and 18.1% (two out of eleven rats) in the PILO + MDIVI group. However, the z-test for rates and proportions revealed no statistically significant differences between the groups (*p* = 0.739), indicating that Mdivi-1 administration did not significantly affect the associated mortality. Figure 2A shows the Kaplan–Meier curve representing the survival probability throughout the experimental procedure for pilocarpine-treated rats.

### 3.2. Overall BW Changes

The percentage of BW changes is presented in Figure 2B. When compared to the BW of experimental day −1, and as expected, SE induced a significant loss of approximately 15–20% of BW in both the PILO + VEH and PILO + MDIVI-1 groups (*p* = 0.004 for both). When BW changes from the two pilocarpine-injected groups (PILO + VEH vs. PILO + MDIVI-1) were compared between them, no statistically significant differences were found, suggesting that the weight loss induced by SE was not affected by Mdivi-1 administration. Likewise, in non-insulted rats, Mdivi-1 had no effects on BW.

### 3.3. Brain Glucose Metabolism Assessed by [^18^F]FDG PET

#### 3.3.1. VOI-Based Quantitative Analysis (SUV Measurements)

**Figure 3:**
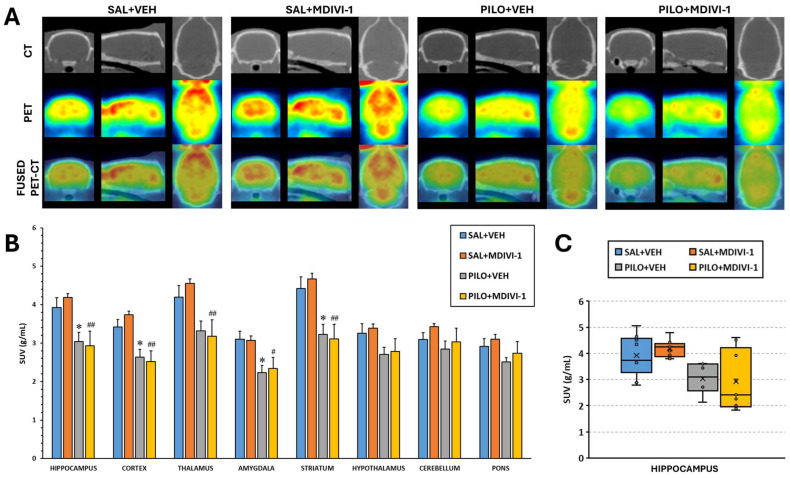
Three days after SE was induced by lithium–pilocarpine, acute brain hypometabolism was significant in all areas measured, except for the hypothalamus, cerebellum, and pons. Mdivi-1 had no effects. (**A**). Illustrative coronal, sagittal, and trans-axial images depicting [^18^F]FDG uptake in the four experimental groups. First row: representative CT images. Second row: [^18^F]FDG PET images. Third row: [^18^F]FDG PET/CT merged images. (**B**) [^18^F]FDG uptake by the different brain areas is shown as SUV units (means ± SEMs, n = 7–10 rats/group); * *p* < 0.05 vs. SAL + VEH; # *p* < 0.05 vs. SAL + MDIVI-1; ## *p* < 0.001 vs. SAL + MDIVI-1. (**C**) Box-and-whisker plot illustrating the distribution of hippocampal SUV values. The first and third quartiles (Q1 and Q3) were computed using the exclusive method, which excludes the median from the calculation.

The analysis of SUV data from [^18^F]FDG PET imaging revealed that SE induced by pilocarpine led to significant glucose hypometabolism in multiple brain regions, regardless of Mdivi-1 treatment. In the PILO + VEH group, significant reductions in [^18^F]FDG uptake were observed in the hippocampus (*p* = 0.04), cortex (*p* = 0.018), amygdala (*p* = 0.014), and striatum (*p* = 0.012). Similarly, the PILO + MDIVI-1 group showed significant hypometabolism in the hippocampus (*p* = 0.002), cortex (*p* < 0.001), thalamus (*p* = 0.003), amygdala (*p* = 0.02), and striatum (*p* < 0.001) (Figure 3A,B). No significant differences were observed between the PILO + VEH and PILO + MDIVI-1 groups across any of the regions studied via VOI-based analysis.

#### 3.3.2. Voxel-Wise Analysis (SPM)

**Figure 4:**
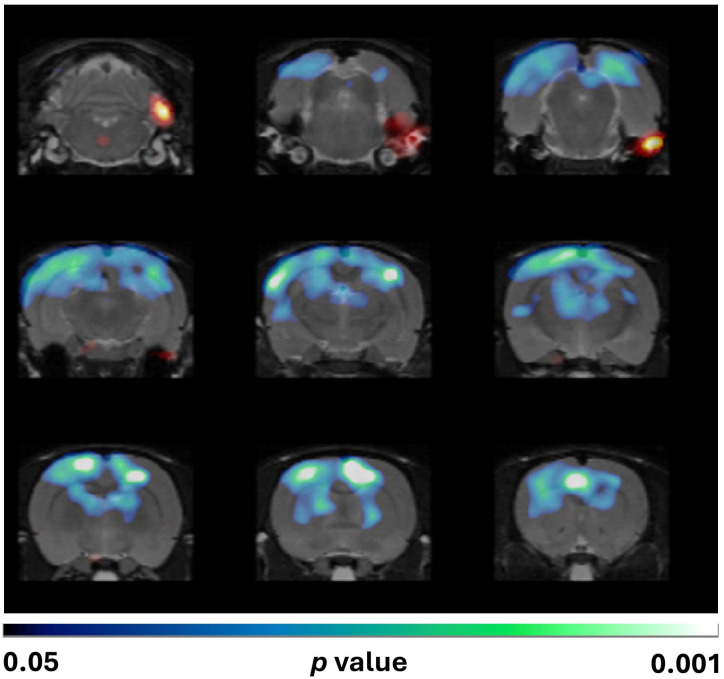
SPM images obtained after normalization to whole-brain [^18^F]FDG uptake and comparing the PILO + VEH group with the PILO + MDIVI-1 group. The corresponding images are shown overlaid on a rat T2-MRI template in coronal view from the striatum (**bottom right panel**) to the cerebellum (**upper left panel**). As observed in SPM images, Mdivi-1 treatment significantly enhanced the hypometabolism in some hippocampal and cortical areas. Color scale: color code bar for clusters showing statistically significant hypometabolism. Results are shown at *p* < 0.05 (uncorrected for multiple comparisons).

However, the SPM analysis of [^18^F]FDG uptake normalized to the whole brain revealed that Mdivi-1 administration exacerbated SE-induced hypometabolism compared to the vehicle-treated group. Noticeably, the brain regions showing enhanced hypometabolism were cortical areas (predominantly within the frontal, parietal, and occipital lobes) and the dorsal hippocampus (Figure 4).

### 3.4. Histopathological Evaluation: Neurodegeneration

**Figure 5:**
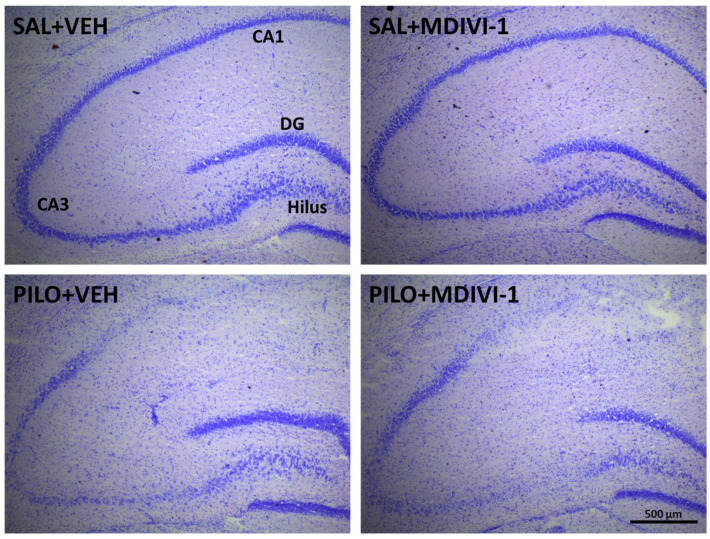
Representative micrographs of Nissl (cresyl violet) staining illustrating the neuronal death in the anterior hippocampus 3 days after the lithium–pilocarpine-triggered SE. As qualitatively observed, the disruption of hippocampal integrity was not affected by Mdivi-1 administration. Scale bar: 500 µm.

Cresyl violet staining revealed visible neuronal loss in the hippocampal subregions CA1, CA3, and the hilus in both SE groups. No apparent neuroprotective effect of Mdivi-1 was observed (Figure 5).

**Figure 6:**
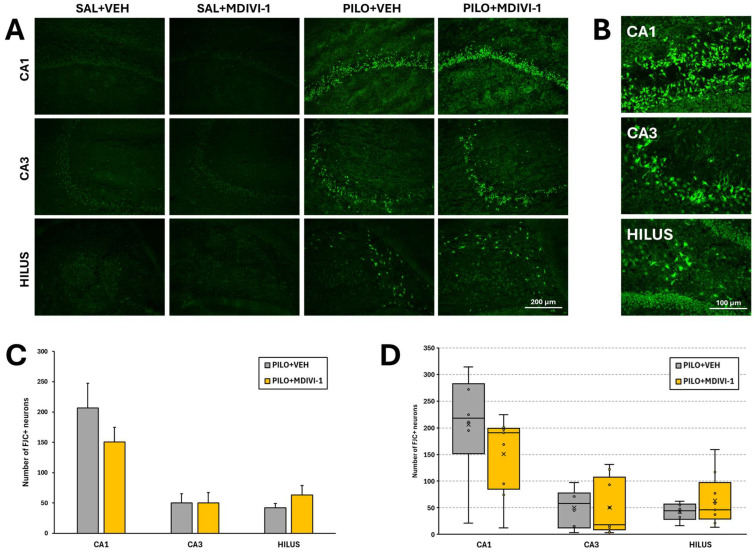
Hippocampal neurodegeneration evaluated 3 days after SE was not affected by Mdivi-1, as evaluated by FJC staining. (**A**) Representative images (10×) at the level of the CA1 (top row), CA3 (mid row), and dentate gyrus/hilus (bottom row) in the 4 experimental groups. Scale bar: 200 µm. (**B**) Magnified images (20×) showing FJC-labeled neurons induced by pilocarpine in the 3 hippocampal subregions. Scale bar: 100 µm. (**C**) Bar plot corresponding to the numbers of degenerating neurons in the hippocampus of SE-insulted rats (means ± SEMs; n = 7–10 rats/group; rats that survived the experimental procedure). No significant differences between the PILO + VEH and PILO + MDIVI-1 groups were found (Student’s *t*-tests). (**D**) Box-and-whisker plot illustrates the distribution of FJC+ cells in the three hippocampal subregions. The first and third quartiles (Q1 and Q3) were computed using the exclusive method, which excludes the median from the calculation.

The Nissl findings were further supported by FJC staining. Quantification of FJC-positive neurons demonstrated significant neuronal degeneration in PILO + VEH rats: CA1 (206.3 ± 41.1), CA3 (50.3 ± 14.7), and hilus (42.3 ± 6.8). As shown, similar patterns were observed in PILO + MDIVI-1 rats: CA1 (150.9 ± 24.2), CA3 (50.1 ± 17.2), and hilus (63.0 ± 15.9), with no statistically significant differences between the SE groups (Figure 6A–C). As expected, no neurodegeneration was detected in the hippocampus of control SAL + VEH or SAL + MDIVI-1 rats.

### 3.5. Astrocyte Reactivity (GFAP Immunofluorescence Staining)

**Figure 7:**
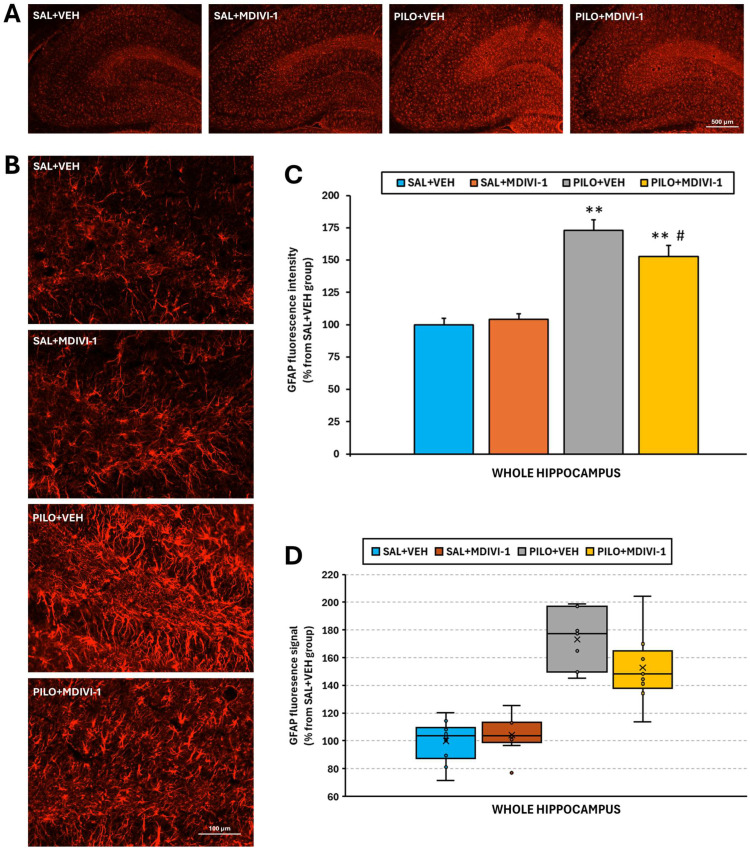
Mdivi-1 reduced reactive astrocyte reactivity 3 days after SE, as assessed by GFAP immunofluorescence staining. (**A**) Representative images (4×) of the whole hippocampus corresponding to the 4 experimental groups. Scale bar: 500 µm. (**B**) Magnified images (20×) of the hilus/dentate gyrus area. Scale bar: 100 µm. (**C**) Bar plot corresponding to the fluorescence quantified in the hippocampus (means ± SEMs; n = 7–10 rats/group; rats that survived the experimental procedure). ** *p* < 0.001 vs. their respective control non-insulted groups; # *p* < 0.05 indicates differences between the PILO + VEH and PILO + MDIVI-1 groups. (**D**) Box-and-whisker plot illustrating the values of the GFAP+ hippocampal fluorescence signal in the four experimental groups. As before, the first and third quartiles (Q1 and Q3) were computed using the exclusive method, which excludes the median from the calculation.

SE resulted in a marked increase (~70%, *p* < 0.001) in the GFAP fluorescence intensity in the hippocampus of PILO + VEH rats compared to SAL + VEH rats, indicating robust astrocyte activation (Figure 7A–C). While Mdivi-1 did not prevent the increase in GFAP expression, it significantly attenuated the magnitude of astrogliosis. Specifically, the GFAP intensity in PILO + MDIVI-1 rats was approximately 50% higher than in SAL + MDIVI-1 rats (*p* < 0.001), but ~20% lower than in PILO + VEH rats (*p* < 0.05). Morphologically, SE induced astrocyte hypertrophy, including thickened cell bodies and processes, which were slightly less pronounced in the MDIVI-1-treated group.

### 3.6. Microglia-Mediated Neuroinflammation ([^3^H]PK11195 Autoradiography)

**Figure 8:**
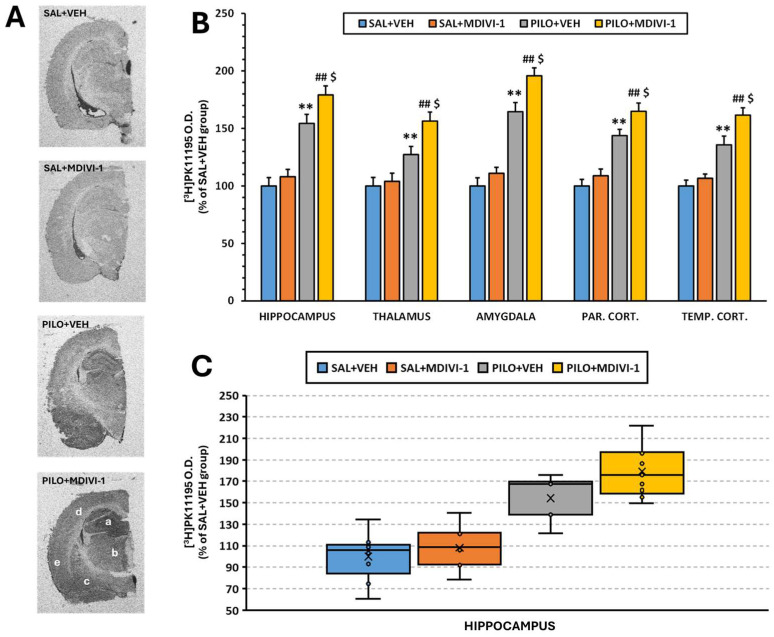
Mdivi-1 treatment exacerbated microglia-induced neuroinflammation 3 days after SE, as measured by [^3^H]PK11195 binding in major brain regions involved in epileptogenesis. (**A**) Representative [^3^H]PK11195 autoradiograms of the 4 experimental groups at the level of the anterior hippocampus. The letter code corresponds to the following brain regions: a, hippocampus; b, thalamus; c, amygdala; d, parietal cortex; and e, temporal cortex. (**B**) [^3^H]PK11195 binding expressed in O.D. as a percentage of the SAL + VEH group. The data are shown as means ± SEMs (n = 7–10 rats/group; rats that survived the experimental procedure). ** *p* < 0.001 PILO + VEH vs. SAL + VEH; ## *p* < 0.001 PILO + MDIVI-1 vs. SAL + MDIVI-1 and $ *p* < 0.05, PILO + MDIVI-1 vs. PILO + MDIVI-1, two-way ANOVA followed by the post hoc Tukey test. (**C**) Box-and-whisker plot showing the autoradiographic signal (O.D.) at the level of the hippocampus in the four experimental groups. The first and third quartiles (Q1 and Q3) were calculated using the exclusive method, which excludes the median from the calculation.

Autoradiographic analysis using [^3^H]PK11195 as a marker of microglial activation revealed that Mdivi-1 had no effect on basal neuroinflammation levels in non-injured rats. In contrast, SE caused a significant increase in optical density (O.D.) across all examined regions compared to the SAL + VEH treatment (*p* < 0.001; Figure 8A,B). This increase was also observed in the PILO + MDIVI-1 group (*p* < 0.001). Notably, Mdivi-1 treatment further exacerbated SE-induced neuroinflammation compared to the PILO + VEH group (*p* < 0.05), suggesting that in this context, Mdivi-1 may intensify the neuroinflammatory response mediated by microglia.

## 4. Discussion

The present study has investigated the impact of Mdivi-1 administration following pilocarpine-induced SE on early brain glucose metabolism, hippocampal neuronal death and neurodegeneration, and glia-mediated neuroinflammatory responses.

The lithium–pilocarpine rat model of SE is a widely used and well-characterized experimental paradigm to study TLE and epileptogenesis [10]. It resembles many, but not all, pathological features of human TLE [8,9]. To date, few reports have attempted to study the effects of Mdivi-1 or altered Drp1 mitochondrial dynamics on the lithium–pilocarpine model of SE in rats, and in all cases, Mdivi-1 was administered before SE [23,24,25,26,27].

In the lithium–pilocarpine model, SE is followed by a latent phase marked by a widespread reduction in glucose brain metabolism, which is recognized as an early indicator of epileptogenesis [13,15,16,17,18,42,44,45,46]. This metabolic decline coincides with extensive neuronal damage, cell death, inflammation, and pronounced reactive gliosis involving both astrocytes and microglia [13,14,16,17,19,47]. These pathological changes eventually culminate in a chronic epileptic condition defined by the occurrence of SRS. Therefore, we chose this silent period (3 days after SE) to study the [^18^F]FDG PET and neurohistochemical changes. Consistent with prior work, our results confirm the characteristically described features, further reinforcing the reliability of the lithium–pilocarpine model in mimicking early epileptogenic processes.

Surprisingly, Mdivi-1 treatment, despite effectively reducing GFAP expression, failed to mitigate metabolic or histological damage, and instead exacerbated cortical hypometabolism and microglia-mediated neuroinflammation. These findings suggest that acute suppression of astrocyte reactivity, though generally considered beneficial, may in fact be detrimental when mistimed or applied without cell-type specificity.

Mdivi-1 is widely used as a pharmacological tool and is particularly effective at modulating mitochondria-dependent stress responses in astrocytes by inhibiting Drp1 [32,33,48]. Inhibition of excessive mitochondrial fission by Mdivi-1 has been shown to exert cytoprotective effects on age-related diseases [49]; animal models of neurodegenerative diseases, such as Alzheimer’s disease, Parkinson’s disease, and multiple sclerosis [22]; as well as different animal models of epileptogenesis [23,24,25,26,27,28]. However, here, it is important to notice that in those studies, Mdivi-1 was always administered preventively before the insult.

In our study, we did not specifically assess Drp1 expression in astrocytes, and so we cannot confirm whether in our experiment SE induced changes in Drp1 levels in this cell population. Nevertheless, numerous studies have provided compelling evidence that collectively underscore the relevance of Mdivi-1 as a modulator of astrocyte reactivity and mitochondrial dynamics in various neuropathological contexts, including neurodegeneration, trauma, ischemia, and epileptogenesis [35,50,51,52].

It is also worth highlighting that accumulating evidence indicates significant limitations that warrant some caution when interpreting the effects of Mdivi-1. Regarding the latter, Mdivi-1 lacks cellular selectivity. It affects not only astrocytes but also neurons, microglia, and peripheral cell types [34]. Its molecular specificity has been challenged. Thus, although initially described as a selective Drp1 inhibitor [35], subsequent studies have shown that Mdivi-1 can exert Drp1-independent effects, including direct inhibition of mitochondrial complex I, leading to decreased mitochondrial respiration and an altered redox status [34,36]. Moreover, not only does the effective range of Mdivi-1 doses vary widely across different models but the in vivo pharmacokinetics are yet to be well defined. At high concentrations, Mdivi-1 may induce off-target effects, including mitochondrial depolarization or apoptosis, depending on the cell type and context [53,54]. In the case of astrocytes, which rely on mitochondrial dynamics to support metabolic and homeostatic functions, non-specific mitochondrial perturbations may inadvertently disrupt astrocytic metabolism and signaling pathways, potentially confounding interpretations that attribute changes in astrocyte reactivity solely to Drp1 inhibition [32]. Altogether, the lack of cellular and molecular specificity, and the timing, doses and routes of administration might be crucial determining the overall effects of Mdivi-1.

In our study, Mdivi-1 was i.p. administered 1 min and 24 h after SE onset at a dose of 1.25 mg/kg dissolved in DMSO. Here, it is worth noting that the dose and route of administration were chosen based on previously reported data showing the neuroprotective effects of Mdivi-1 on the lithium–pilocarpine model of SE. In the lithium–pilocarpine model of SE, Luo et al. (2020) reported that Drp1 expression was significantly upregulated in the hippocampus and temporal neocortex following SE, and that its localization was predominantly neuronal [25]. In the same study, Mdivi-1 administered at a dose of 1.2 mg/kg, i.p., 30 min before pilocarpine was shown to be effective at reducing Drp1 expression and improving mitochondrial ultrastructure in CA1 neurons, delaying the seizure onset and reducing the severity of the seizures [25]. Additionally, i.v. bolus of Mdivi-1 (1.25 mg/kg) given 15 min before pilocarpine administration significantly attenuated hippocampal neuronal death measured 3 days after SE [24]. Furthermore, these authors showed that this neuroprotective effect was dose-dependent and was not observed when Mdivi-1 was administered at a dose of 0.25 mg/kg.

Although Mdivi-1 is usually dissolved in DMSO and can cross the blood–brain barrier (BBB), with an estimated half-life of 12 h [55], the effective dose range and route of administration vary widely across studies and models. Moreover, the in vivo pharmacokinetic properties of Mdivi-1 remain poorly defined.

In the current study, Mdivi-1 administration neither significantly affected the mortality rate nor the overall BW loss that typically are observed as consequences of the severity of the SE induced by pilocarpine. Likewise, we did not find any significant neuroprotective effect based on hippocampal signs of neurodegeneration and neuronal death (Figure 5 and Figure 6). These results suggest that the timing of Mdivi-1 administration, either before or after SE, is key in determining its potential protective effects.

The present study provides novel insights into the metabolic consequences of Mdivi-1 administration following SE induced by lithium–pilocarpine in rats. Mdivi-1 administered shortly after SE onset (1 min and 24 h later) failed to attenuate the SE-induced metabolic impairment when the analysis of PET imaging data was conducted by the conventional VOI method (Figure 3). Furthermore, the voxel-by-voxel analysis by SPM normalized to whole-brain [^18^F]FDG uptake revealed that Mdivi-1 induced a more pronounced and spatially distinct glucose hypometabolism, particularly in some cortical and hippocampal areas (Figure 4). This difference between these methods of analysis highlights the ability of SPM for detecting regional changes without requiring a priori anatomical assumptions. Unlike the VOI-based analysis, SPM evaluates the whole brain voxel by voxel, allowing for the detection of subtle or unexpected effects. In addition, normalization to whole-brain activity reduces confounding from peripheral tracer uptake and global cerebral blood flow alterations.

The marked hypometabolism observed in limbic–cortical regions is of particular relevance, given that the functional connectivity of these areas, which are involved in executive, cognitive, sensory and emotional processing, is disrupted in epilepsy [56,57]. Several mechanisms may be associated with this effect. For example, mitochondrial dysfunction in the surviving cortical neurons due to impaired fission and turnover of damaged organelles by Mdivi-1 might have led to bioenergetic deficits. Additionally, differential effects of Mdivi-1 on neurons vs. glial cells might have altered the overall [^18^F]FDG uptake profile, masking or amplifying specific metabolic patterns. It is also interesting that the exacerbated hypometabolism, particularly in hippocampal and cortical areas, might indicate perturbed synaptic activity and connectivity in these areas [58]. In this context, Mdivi-1 administered by i.p. injection before SE induction in rats has been shown to increase the latency to the first seizure, to reduce the number of epileptic seizures, and to reduce the effects of SE on increasing Drp1 expression in the hippocampus and cortex [25]. Furthermore, there is robust and consistent evidence that Mdivi-1, when administered before damage, protects cortical neurons in epilepsy models, including the pilocarpine model of SE [25,59], and preserves mitochondrial health and reduces neuronal cell death in different types of brain injury, such as ischemia/stroke [60], glutamate-mediated neurotoxicity [29,61], and traumatic brain injury [62,63]. Thus, Mdivi-1 clearly confers protective and restorative effects on both the cortex and hippocampus by inhibiting Drp1-mediated mitochondrial fission. While the neuronal benefits of Mdivi-1 are well-documented in cortical neurons, astrocyte-specific effects in the cortex remain understudied. It is particularly intriguing that Mdivi-1, which is widely recognized for its neuroprotective effects when administered preventively in various experimental models, produced adverse outcomes in the present study when applied after the onset of SE. This discrepancy is unlikely to stem from the use of the model itself, as numerous studies have reported beneficial effects of post-SE administration of different antiepileptic drugs on this same model [64,65]. Interestingly, a previous study by Córdova-Dávalos and coworkers using the lithium–pilocarpine model demonstrated that SE promotes mitochondrial fusion [27]. This raises the possibility that SE had induced a shift toward mitochondrial fusion, as reported [27], thus creating an imbalance in fusion/fission dynamics that could explain the divergent effects of Mdivi-1 depending on the timing of administration. In this scenario, inhibiting fission after SE, when fusion is already predominant, might further disrupt mitochondrial dynamics, impair recovery, and even result in detrimental effects.

To our knowledge, this is the first study employing [^18^F]FDG PET to assess the impact of Mdivi-1 administration on brain glucose metabolism. Considering that cerebral glucose hypometabolism has been proposed as an early biomarker of epileptogenesis in several models [15,66], our current findings raise important questions. The lack of effects of Mdivi-1 on improving the metabolic deficits and/or its effect on intensifying the hypometabolism in cortical areas might indicate either no beneficial effects or potential deleterious effects, at least when administered during the early post-SE period. However, it is essential to acknowledge that this study did not evaluate long-term outcomes such as the frequency or severity of SRS, neurocognitive function, or chronic metabolic profiles. Consequently, while the data suggest that Mdivi-1 may disrupt early metabolic recovery after SE, we cannot conclude whether these effects might have a predictive value in epileptogenesis or simply reflect an altered acute-phase response.

In the lithium–pilocarpine model, glial cells—particularly astrocytes and microglia—not only respond to the initial SE insult but play crucial and tightly interconnected active roles in the initiation and progression of neuroinflammation, neurodegeneration, and network reorganization [67,68,69]. Reciprocal astrocyte–microglia interactions are emerging as critical regulators of the neuroinflammatory cascade and circuit remodeling during epileptogenesis.

Previously reported studies have shown that Mdivi-1 treatment is effective at reducing astrocyte reactivity in different types of neurological injury [32,33,52]. Thus, one of our main objectives was to evaluate the contribution of astrocytes to SE-induced damage by inhibiting their reactivity using Mdivi-1. Our results clearly showed that Mdivi-1 reduced astrocyte reactivity in response to SE, as evidenced by the reduced GFAP expression (Figure 7). However, as previously indicated, this reduction was neither translated into neuroprotection nor did it rescue the overall brain metabolic deficits, but, based on the SPM analysis, it might have exacerbated cortical hypometabolism. Furthermore, the inhibition of astrocyte reactivity by Mdivi-1 exacerbated the microglial activation in SE-insulted rats.

Following SE, astrocytes are among the first cells to respond, exhibiting rapid morphological and transcriptional changes commonly referred to as reactive astrogliosis. This reactive state, including the upregulation of GFAP, is also accompanied by altered potassium and glutamate buffering, and by the release of cytokines and ATP, which in turn modulate microglial activity [70,71]. In the lithium–pilocarpine model, astrocyte activation occurs rapidly after SE, with the upregulation of GFAP within the first 72 h post-SE [13,14,16,17,19]. Astrocyte reactivity generally precedes or coincides with microglial activation, suggesting a temporal hierarchy in glial responses [72]. Several studies have shown that astrocyte-derived signals can functionally regulate microglial activation, either promoting or limiting inflammatory responses, depending on the timing and context [73]. Microglial cells, in turn, secrete cytokines that further influence astrocyte reactivity, establishing a reciprocal regulatory loop [74,75]. This temporal sequence is critical.

In this context, our current results show that when acute astrocyte reactivity is pharmacologically attenuated by Mdivi-1, microglia activation measured 3 days after SE becomes hyperactive, suggesting a complex and possibly inhibitory effect of astrocytes on microglia during the early post-SE phase. As previously reported [32,33], this is likely due to the loss of astrocyte-derived anti-inflammatory or homeostatic signals in the early stages of epileptogenesis. Thus, our results reinforce the hypothesis that astrocytes might exert an acute regulatory “braking” effect on microglial activation.

In the lithium–pilocarpine model, astrocyte and microglial reactivity remain elevated weeks to months post-SE, particularly in hippocampal and limbic regions [15,72]. Astrocyte dysfunction may drive ongoing microglial activation, and vice versa, with astrocyte-derived mediators serving as long-term microglial stimuli [76,77,78,79]. Recent evidence suggests that glial communication becomes increasingly dysregulated in chronic epilepsy, potentially establishing a positive feedback loop of gliosis and inflammation [80]. This includes epigenetic modifications in astrocytes and microglia that maintain their reactive states independent of external stimuli [81].

Here, it is interesting to notice that the astrocyte–microglia crosstalk is a shared feature of epileptogenesis found in other chemical models of SE, such as the kainic acid [15,74,82,83,84,85] and electrically induced models of SE [86]. Nevertheless, the directionality, timing, and functional consequences of this interaction are model-dependent.

Then, our results from the lithium–pilocarpine model of SE align with reports showing that astrocyte–microglia crosstalk is temporally dynamic and supports the context-dependent factor [80]. Thus, in the early epileptogenic window, astrocytes seem to act by restraining microglial responses, and consequently, the early inhibitory intervention with Mdivi-1 may have disrupted the neuron–astrocyte–microglia homeostasis, resulting in worse outcomes.

Our results provide novel insights showing that Mdivi-1 failed to attenuate the SE-induced metabolic impairment; on the contrary, it exacerbated glucose hypometabolism, especially in cortical regions. Cerebral glucose hypometabolism has been proposed as an early biomarker of epileptogenesis in various animal models [12,45,46,66,87], and the fact that Mdivi-1 intensified cortical hypometabolism could indicate a deleterious effect—at least when administered during the early post-SE period. However, it is essential to acknowledge that this study did not evaluate other long-term outcomes, such as the frequency or severity of spontaneous seizures, neurocognitive function, or chronic metabolic profiles.

While our study is subject to some limitations, it also opens up new avenues of inquiry that warrant further investigation in future studies. First, the lack of mechanistic insight into why Mdivi-1, which is typically neuroprotective in preventive models, exacerbates pathology when administered post-SE remains unresolved. This study does not assess the expression of mitochondrial fusion/fission proteins, which could elucidate the observed effects. The determination of these fusion/fission proteins in post-SE brains of animals treated or not treated with Mdivi-1 may be worthwhile in future studies. Second, increased GFAP expression is a hallmark of reactive astrocytes in epileptic foci, including the sclerotic hippocampus. Astrocyte reactivity induced by lithium–pilocarpine has been also evidenced by increases in the levels of other proteins, such as glutamine synthetase (GS) and S100B, among others [88]. Thus, it seems that the status of GS+ astrocytes, those expressing glutamine synthetase, would be functionally more relevant to epileptogenesis, specifically during the latent phase, as shown in rats injected with kainic acid [89]. Furthermore, evidence suggests that GS reactivity increases during the latent phase of epilepsy and that in patients with advanced temporal lobe epilepsy (TLE), the loss of GS+ astrocytes, rather than changes in GFAP+ astrocytes, correlates with neuronal degeneration [90]. Therefore, the observed reduction in GFAP reactivity following Mdivi-1 treatment may not necessarily reflect a beneficial modulation of epileptogenic processes. This underscores the need for future studies to assess GS expression and function directly, as relying solely on GFAP as a marker may overlook critical aspects of astrocyte-mediated pathology because GS+ astrocytes are more functionally relevant to early and later stages of the epileptogenesis process. Likewise, the analysis of morphological parameters such as the astrocyte size or thickened processes would provide more detailed information regarding astrocyte reactivity.

Third, the use of DMSO as a solvent raises concerns about drug delivery efficacy and potential confounding effects, suggesting that alternative solvents and administration routes should be considered.

Although behavioral (by the Racine scale) and histological outcomes were evaluated, the absence of EEG data limits our ability to characterize seizure dynamics and the neurophysiological impact of Mdivi-1. Future studies incorporating EEG monitoring will provide a more comprehensive understanding of its therapeutic antiseizure potential. Likewise, we did not evaluate eventual changes in proinflammatory cytokine expression or the levels of mitochondrial dynamics proteins, such as Drp1, Mfn1/2 and OPA1, in post-SE brains treated with Mdivi-1, which could provide mechanistic insights into the differential effects of Mdivi-1 depending on the timing of administration. Altogether, future pharmacokinetic, EEG, and mechanism-focused studies are needed to better define the concentration–effect relationship and specificity of Mdivi-1 in brain tissue.

Finally, yet of equal importance, we must acknowledge that we have studied the lithium–pilocarpine model of SE in adult male rats, a model with intrinsic characteristics that are difficult to generalize to other models of SE [10]. Consequently, the observed adverse effects of post-SE administration of Mdivi-1, including exacerbated cortical hypometabolism and altered glial reactivity, may not necessarily translate to other experimental paradigms. Future studies employing diverse models of acute seizures and epileptogenesis are needed to determine whether the timing-dependent effects of Mdivi-1 are consistent across different contexts.

In summary, our data did not show neuroprotective effects of Mdivi-1 when administered shortly after SE. Additionally, Mdivi-1 not only did not rescue brain glucose hypometabolism but also, when analyzed by SPM, it showed disruptive effects after SE, particularly in cortical regions. Furthermore, Mdivi-1 reduced astrocyte reactivity and exacerbated microglia activation, supporting a potential regulatory role of astrocytes in inhibiting early microglia-mediated neuroinflammation. Thus, our study suggests that the acute blockade of astrocyte reactivity may lead to an unintended short-term pro-inflammatory state mediated by hyperactive microglia in the early phase of epileptogenesis. This shift might counteract any potential neuroprotective effect and the ability of Mdivi-1 to rescue brain metabolic deficits. Even though these effects might simply reflect altered acute-phase responses, we cannot discard the possibility that they might have long-term deleterious consequences by exacerbating epileptogenesis in later phases.

## 5. Conclusions

In summary, astrocyte and microglia interactions in the lithium–pilocarpine rat model of SE are functionally interdependent and temporally coordinated, shaping the inflammatory milieu. While Mdivi-1 has shown promise in preventing brain damage in different types of neurological disorders, including epilepsy, our findings challenge the assumption that the inhibition of mitochondrial fission is always beneficial in post-SE models. Furthermore, our results reflect the need for determining the temporally precise conditions of Mdivi-1 administration and they also emphasize the complexity of targeting mitochondrial dynamics in epilepsy.

Future studies should use conditional astrocyte-specific genetic models to determine whether astrocyte reactivity might be highly interesting for the early containment of microglial activation. Longitudinal profiling of astrocytic modulation beyond the acute phase might be essential to determine how early glial interventions shape long-term neuroprotection, seizure susceptibility, and cognitive outcomes.

## Data Availability

Data will be available upon reasonable request to the corresponding author.

## Abbreviations

The following abbreviations are used in this manuscript:

[^18^F]FDG	2-Deoxy-2-[^18^F]fluoro-D-glucose
BW	Body weight
CT	Computed tomography
DMSO	Dimethyl sulfoxide
Drp1	Dynamin-related protein 1
EEG	Electroencephalography
FBP	Filtered back projection
FJC	Fluoro Jade C
GFAP	Glial fibrillary acidic protein
GS	Glutamine synthetase
Mdivi-1	Mitochondrial division inhibitor-1
MLEM	Maximum likelihood expectation maximization
MRI	Magnetic resonance imaging
O.D.	Optical density
PET	Positron emission tomography
SE	Status epilepticus
SEM	Standard error of the mean
SPM	Statistical parametric mapping
SRS	Spontaneous recurrent seizures
SUV	Standardized uptake value
TLE	Temporal lobe epilepsy
TSPO	18 kDa translocator protein
VOI	Volume of interest
